# European primary datasets of alien bacteria and viruses

**DOI:** 10.1038/s41597-022-01485-1

**Published:** 2022-07-13

**Authors:** Chiara Magliozzi, Marc Artois, Assunta Bertaccini, Thierry Candresse, Konstantinos Tsiamis, Fabio D’Amico, Ivan Deriu, Eugenio Gervasini, Ana Cristina Cardoso

**Affiliations:** 1 European Commission, Joint Research Centre (JRC), Ispra, Italy; 2 LISAE (Lorraine investigation in animal and environmental health), Lagney, France; 3 Alma Mater Studiorum, University of Bologna, Bologna, Italy; 4 Univ.Bordeaux, INRAE, UMR 1332 BFP, Villenave d’Ornon, France

**Keywords:** Invasive species, Microbial ecology

## Abstract

Bacteria and viruses are a natural component of Earth biodiversity and play an essential role in biochemical and geological cycles. They may also pose problems outside their native range, where they can negatively impact on natural resources, wildlife, and human health. To address these challenges and develop sustainable conservation strategies, a thorough understanding of their invasion related- factors is needed: origin, country and year of introduction, and pathways dynamics. Yet, alien bacteria and viruses are underrepresented in invasion ecology studies, which limits our ability to quantify their impacts and address future introductions. This study provides primary datasets of alien bacteria and viruses of plants and animals present in the European environment. The datasets contain expert-revised data on 446 taxa and their invasion related- factors across terrestrial and aquatic environments. Taxa information are complemented with spatial occurrences. The datasets provide a basis for collaborative initiatives to improve the collection of alien bacteria and viruses’ data, and a starting point for data-driven conservation practices.

## Abstract (summary)

**Table:** 

Measurement(s)	bacteria and viruses in Europe and their definition of alien status
Technology Type(s)	literature review • taxonomic expertise
Sample Characteristic - Organism	bacteria • viruses
Sample Characteristic - Environment	terrestrial • marine• freshwater
Sample Characteristic - Location	Europe

## Background & Summary

Bacteria and viruses are microscopic organisms inhabiting virtually every ecosystem on Earth^1^. They constitute the greatest biodiversity on the planet and play an essential role in global biochemical and geological cycles^2,3^. Despite being an indispensable part of life, bacteria and viruses may cause a range of problems, such as diseases affecting both the health and resources available to e.g., plants, animals, and humans^4–6^. To develop adequate biosecurity and management strategies, it is necessary to know bacteria and viruses’ distributions and link their dispersal dynamics to their region of origin, vectors and impacts on the social-ecological system.

In the last decades, hundreds of bacteria and viruses were introduced in Europe from their native range and called alien species. These introductions were the result of the increasing global transport of commodities, international trade of wildlife species or of their use as biological control agents^7–10^. The alien species (AS) which can establish self-sustaining populations, spread and impact negatively on biodiversity at different biological levels^11–13^ (from single organism to population), and on sustainable exploitation of biodiversity^13^ are called invasive alien species (IAS). In Europe, priority IAS that are introduced accidentally or deliberately into a natural environment are regulated by the Regulation (EU) 1143/2014/EC^14^ (hereafter IAS Regulation), which establishes concerted actions at European Union level to prevent, minimise and mitigate their effects on biodiversity. Invasive alien bacteria and viruses are not considered in the scope of the IAS Regulation, either because they are listed in other legal acts (e.g. Regulation (EU) 2016/2031^15^) or targeted by the human health research^16^. Except for pathogenic (disease-causing) bacteria and viruses, which are the focus of public health epidemiology^17^ (e.g. European Food Safety Authority (EFSA), European Centre for Disease Prevention and Control (ECDC)), the processes of introduction-establishment-invasion of alien (also non-pathogenic) microorganisms have not been yet well investigated, and as a result these groups are poorly represented in alien and invasive alien species databases^18^. In this context, there are three key issues for data paucity on bacteria and viruses. First, their identification is often complex. Unlike many plants and animals, bacteria and viruses are microorganisms difficult to detect without analytical approaches using culture-based or molecular tools^19^. In addition, viruses can be principally detected after recognition of a disease signs (morbidity and mortality)^20^. A second issue is the determination of the organism’s alien status. In invasion science, the definition of alien is based on the evaluation of three main criteria: i) biogeographic barriers, ii) human agency (*i.e*. intentional, unintentional introduction), and iii) survival after introduction^10^. The location and strength of biogeographic barriers is linked to the species or species’ carrier dispersal potential (*e.g*. host, reservoir and vectors) and ecological requirements, and for bacteria and viruses baseline inventories are limited especially across taxonomic realms^18,21,22^. As a result, diagnostic tools such as molecular and phylogenetic analysis are essential to refine the hypothesis of the geographical origin of bacteria and viruses^23^. Finally, it is often a challenge to assign impact to alien bacteria and viruses. They have shorter generation times than plant or animals, and their demographic processes have been little studied in wildlife populations^24^. In this paper, we assembled expert-revised and harmonised datasets of alien bacteria and viruses filling the gap in European biodiversity resources. The datasets give unique insights into taxon traits, invasion-related factors and location characteristics^25^ (*i.e*. taxonomy, year and country of first introduction, native distribution, pathways of introduction, and environment), and invasion processes (*i.e*. introduction, establishment and invasion), starting from the recognition that invaders’ traits interact with invaded communities allowing or preventing invasions. The datasets are not comprehensive of all alien bacteria and viruses in Europe, but focus on those known to affect wild, free-living species of plant and animals. We envision the datasets as an on-going collaborative initiative for easily sharing invasion-related data about alien bacteria and viruses, avoiding duplication of efforts, and as a first step toward knowledge and control of alien pathogens in wild species. The datasets can also be of use in epidemiology, and particularly on zoonoses associated with wildlife, and more broadly by considering alien bacteria and viruses in the practice of invasion science to provide evidence in support of conservation objectives. Finally, we ensure their access openly through the European Alien Species Information Network (EASIN).

## Methods

### Alien status definition

**Fig. 1:**
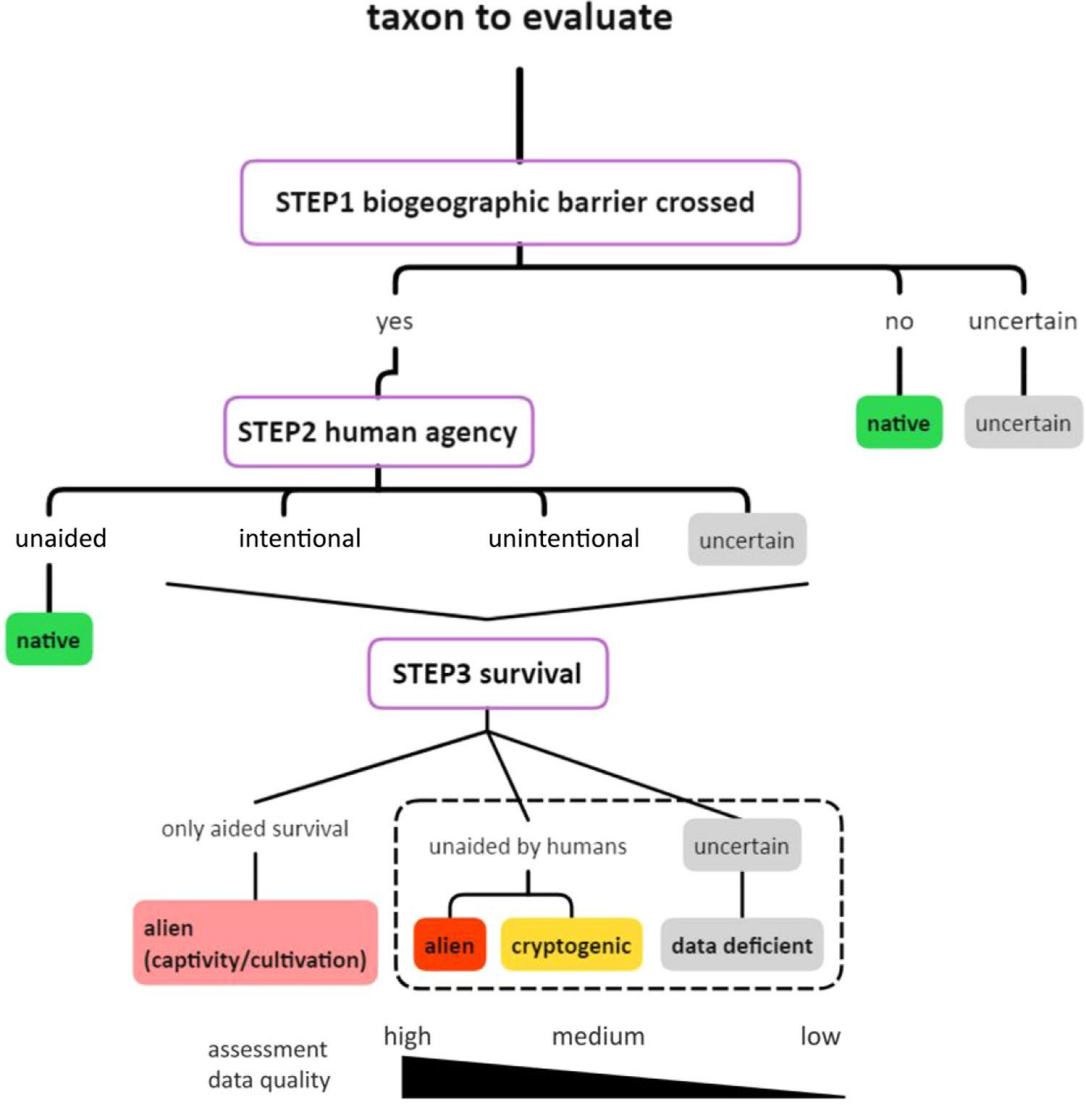
Conceptual scheme to assess the alien status of a taxon. It considers three assessment criteria, *i.e*. biogeographic barriers, human agency, and survival, and the levels of uncertainties of data quality (modified from Essl *et al*.^10^.

The definition of alien species is controversial, and a range of definitions have been put forward over the years^26–29^. For example, in public health epidemiology alien and pathogenic species are named emerging infectious diseases (EIDs^30^) and refer to several mechanisms that allow pathogens to be transmissible to humans, *i.e*. adaptive (genetic changes allowing host jump from animals to humans) and geographical (long-distance or localized translocation). In this paper, we followed the scheme proposed by Essl *et al*.^10^ and applied it to Europe (27 EU Member States, 5 Candidate countries to the European Union, and 32 other neighbouring countries) (Fig. 1). Alien taxa are species or infra-species assessed through a set of criteria, *i.e*. biogeographic barriers, human agency, and survival, considering uncertainties. Alien species as addressed here have crossed biogeographic barriers by direct (intentional) or indirect (unintentional) human agency and survived in the wild (Fig. 1). Following the methodology of Essl *et al*. (2018), the scientific evidence in support of these criteria must be reliable and robust (low uncertainty). Low uncertainty is achieved when the data are of good quality and the assessor estimates a probability of correct classification greater than 90% (Fig. 1). We operationalized the alien species definition by including partly alien species in Europe, a sub-group of the alien taxa with a native distribution in at least one European country (administrative boundaries) but alien in other(s). Our datasets include two additional categories: cryptogenic, *i.e*. species of uncertain alien status, and data deficient, *i.e*. species for which an assessment of the alien status is currently unfeasible because of the lack of data^10^ (Fig. 1, Table 1). Finally, the terms taxon traits, invasion-related factors and location characteristics were adopted from the terminology of Pysek *et al*.^25^ to refer to the species taxonomy, year and country of first introduction, native distribution, introduction pathways, and environment for alien, cryptogenic and data-deficient taxa.

**Table 1:** Number of bacteria and viruses of plant and animals reviewed by experts. The status is assigned according to Fig. 1.

Group	Alien	Cryptogenic	Data Deficient	Total
bacteria	55	17	21	93
viruses	48	19	286	353

### Data collection

Data were retrieved by plants and animal pathologists with expertise on epidemiological surveillance or diagnostics and characterization, including molecular plant/animal-virus interaction and metagenomics. Sources encompassed international scientific and grey literature, disease surveillance databases, repositories, and search engines relevant to epizootiology or epidemiology, ecology and invasion science (Table 2). Additional sources of information are available in the static version of the datasets as.CSV format.

**Table 2:** Examples of bibliographic repositories on bacteria and viruses reviewed by experts.

Database name	Description	Web source link
Centre for Agriculture and Bioscience International (CABI)- Invasive Species Compendium^43^	bacteria, virus	CABI Invasive Species Compendium
European and Mediterranean Plant Protection Organization (EPPO)-EPPO-Q-bank database International Phytoplasmologist Working Group (IPWG)-Phytoplasma Collection	bacteria, plant pests, phytoplasmas	EPPO-Q-bank database; Phytoplasma Collection
World Organisation for animal health (OIE)- WAHIS database	animal pathogens	WAHIS database
Plant Viruses Database –DPV^44,45^	virus	DPV database
Virus Identification Data Exchange (VIDE) project database	virus	VIDE database
Genbank database^46^	virus	integrated through Pubmed
International Committee for the Taxonomy of Viruses (ICTV)	virus	ICTV
National Collection of Plant Pathogenic Bacteria (NCPPB)	bacteria	NCPPB

**Fig. 2:**
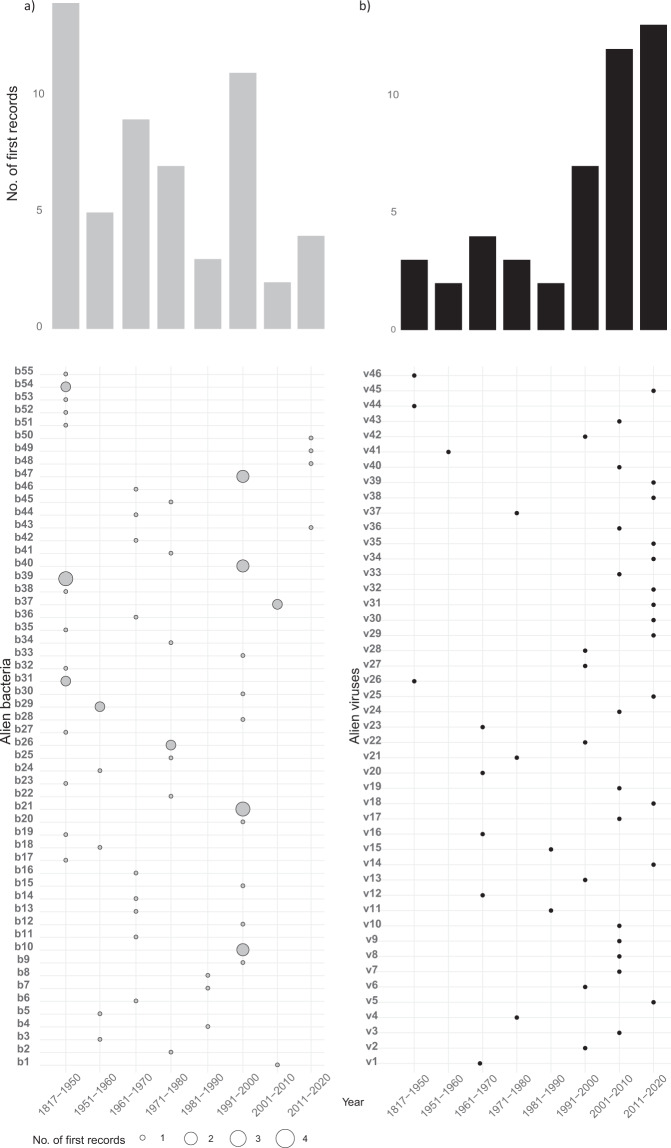
Temporal distribution of first records of alien bacteria (**a**) and viruses (**b**) in European countries (n = 28). Point sizes represent the number of records per species and time period. The Grapevine red blotch virus and the Apple scar skin viroid are not included in the list as the year or the country of introduction in Europe are unknown. See Table 3 for species scientific names and codes in Supplementary File 1.

**Fig. 3:**
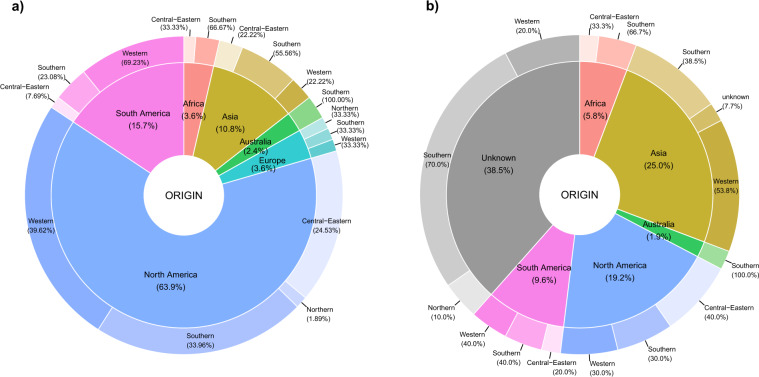
Distribution of alien bacteria (**a**) and viruses (**b**) in European regions according to their origin. The internal pie refers to the Continents of origin (Europe, North America, South America, Asia, Africa, Australia), while the outer circular crown to the European regions invaded. The European regions are grouped into Central and Eastern Europe, Northern Europe, Southern Europe, and Western Europe (EuroVoc). Unknown refers to taxa of unknown origin.

**Fig. 4:**
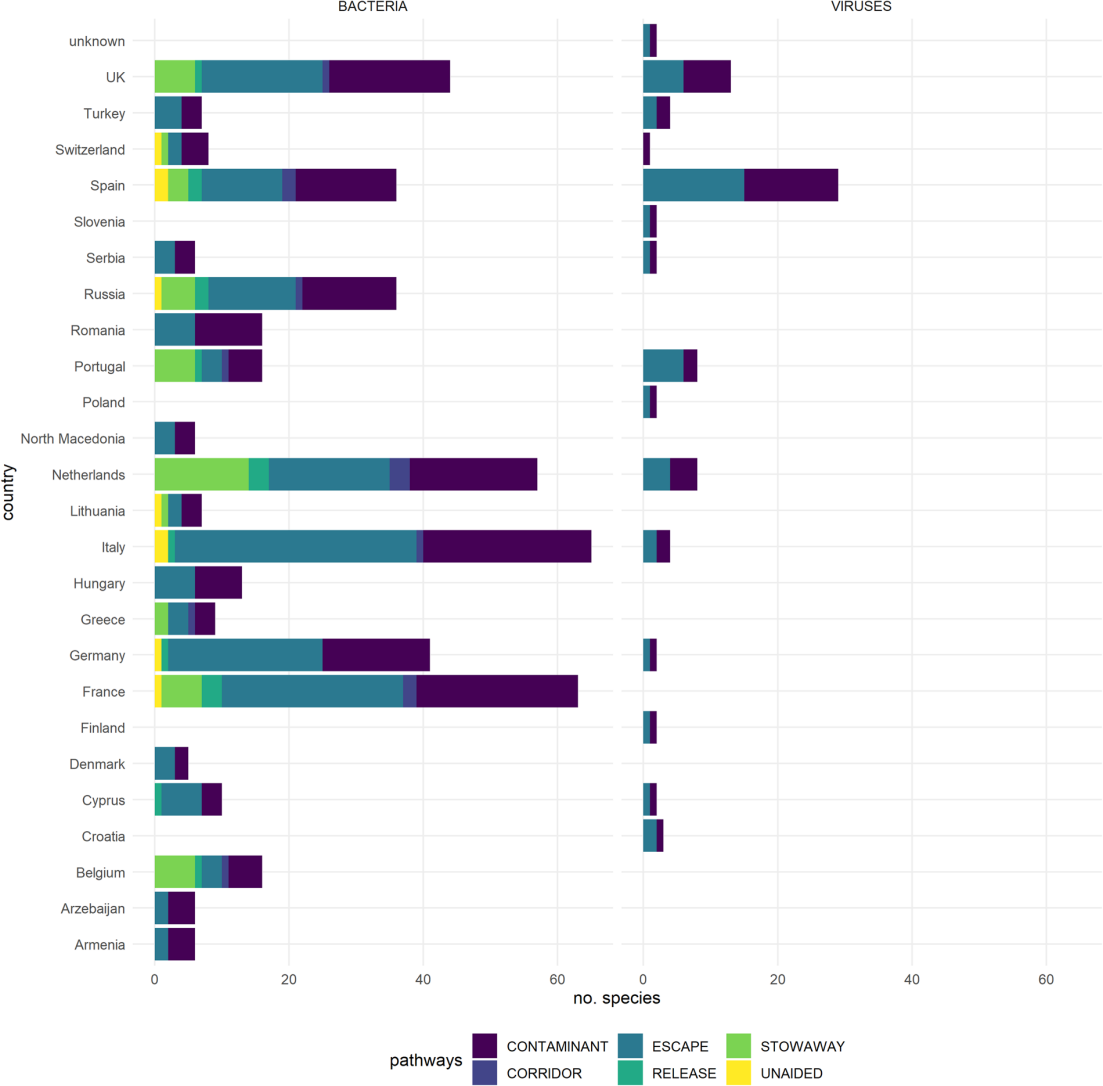
Pathways of first introduction in Europe. Number of species of alien bacteria and viruses and relevant introduction pathway, i.e., contaminant, corridor, escape, release, stowaway, and unaided (Supplementary Table 1), across European countries.

For each species of plant and animal bacteria and viruses the following information is provided: i) valid/accepted nomenclature and taxonomy, ii) the year and country of first introduction, iii) native distribution, iv) primary introduction pathways, iv) environment, and v) spatial reference (Table 3). The taxonomic nomenclature follows the approaches of the Integrated Taxonomic Information System (ITIS) and the European and Mediterranean Plant Protection Organization (EPPO) for bacteria, and the International Committee for the Taxonomy of Viruses’ (ICTV) for viruses. When assigning taxonomy to invasive pathogens, it was not always possible or relevant to address the species level and the ‘strains’ (infra-species taxon) were considered. For phytopathogenic bacteria known as phytoplasmas and liberibacter, the provisional category ‘*Candidatus* Phytoplasma/*Candidatus* Liberibacter’^31^ was used. The year and country of first introduction refers to the first observation of the alien species, and it is often used the name of the first author and the year of publication associated with the source’s primary citation (e.g. bibliographic reference) (Fig. 2). The native distribution data ranges from continent to sub-continent level (administrative boundaries) (Fig. 3), and species with part of their native range in a European country are tagged as partly alien. The first arrival of an alien species in Europe was documented according to the CBD (2014)^32^ pathway categorization scheme: contaminant, corridor, escape, release, stowaway, unaided (Fig. 4, Table 3, Table 1 in Supplementary File 1). The pathways refer to both the host and to the pathogen without the host assigned. A certainty score was given to each assigned pathway for every species. The high certainty (P HIGH) score indicates direct evidence of a pathway. The species is clearly associated to a pathway at the time of introduction to a given locality, and it is often the case of intentional introductions. The medium certainty (P MEDIUM) denotes pathways that can be likely inferred, e.g., the taxon appears for the first time in a locality where a pathway is known to operate. In many cases inference is based on known examples of introductions elsewhere for the same or similar species, the biology and ecology of the species, the habitats and locales it occupies in both the native and introduced range, and its pattern of dispersal (if known). The low certainty (P LOW) is used when not reliable pathways can be assigned to the alien taxon. Inference is based on the activities in the locality where the species was found and may include evidence on similarly behaving species reported elsewhere (Table 3). In cases of unknown level of uncertainty, only the pathway was indicated (P). Finally, taxa spatial records are provided to foster examining the relationship between host and pathogens at different scale, the so-called ‘epidemiological surveillance’^33^.

**Table 3:** Invasion- related factors and location characteristics of the bacteria and viruses datasets. Fields and description of the collected information for each species of bacteria and viruses.

Field	Description
SPECIES SCIENTIFIC NAME	Species scientific name
STATUS	Status of the species, A for Alien, C for Cryptogenic, DD for Data Deficient^10^
ORIGIN	Origin of the terrestrial/freshwater/marine species (Country or Continent). (see Supplementary File 1)
YEAR OF FIRST INTRODUCTION IN EUROPE	Must contain a numeric value (e.g. 2020). For BC dates a minus must be used (e.g. −1500). It is the year of the first observation of an alien species in Europe. It is used as the best available estimate of the year of its initial introduction when the latter could not be determined with certainty, based on a thorough review of the scientific and grey literature.
COUNTRY OF FIRSTINTRODUCTION IN EUROPE	ISO 3166-1 Alpha-2 code of European or neighbouring Country of first introduction (e.g. IT for Italy) including the outermost regions (see Supplementary File 1)
REFERENCE	reference to the publication for the first introduction of the species
DOI	Digital Object Identifier of the publication
NOTES	note about the species introduction
TERRESTRIAL	Value 1 if the species is terrestrial.
FRESHWATER	Value 1 if the species is freshwater.
MARINE	Value 1 if the species is marine.
OLIGOHALINE	Value 1 if the species is estuarine.
PARASITE	Value 1 if the species is parasite
KINGDOM	Taxonomic kingdom of the species
PHYLUM	Taxonomic phylum of the species
CLASS	Taxonomic class of the species
GENOME	Genome composition (optional)
ORDER	Taxonomic order of the species
FAMILY	Taxonomic family of the species
GENUS	Taxonomic genus of the species
REFERENCE	reference to the nomenclature used (e.g. ITIS, EPPO)
PARTLY NATIVE	Value 1 if the species is partly native in Europe.
COUNTRY/Marine Strategy Framework Directive (MSFD) REGION	Value 1 if the species is partly native in such Country or MSFD Region. See Supplementary File 1
CBD 2014 PATHWAYS	Value P_HIGH: primary pathway of introduction with high level of uncertainty.
	Value P_MEDIUM: primary pathway of introduction with medium level of uncertainty.
	Value P_LOW: primary pathway of introduction with low level of uncertainty.
	Value P: primary pathway of introduction with unknown level of uncertainty.
	Value S: secondary pathway of introduction

## Data Records

**Fig. 5:**
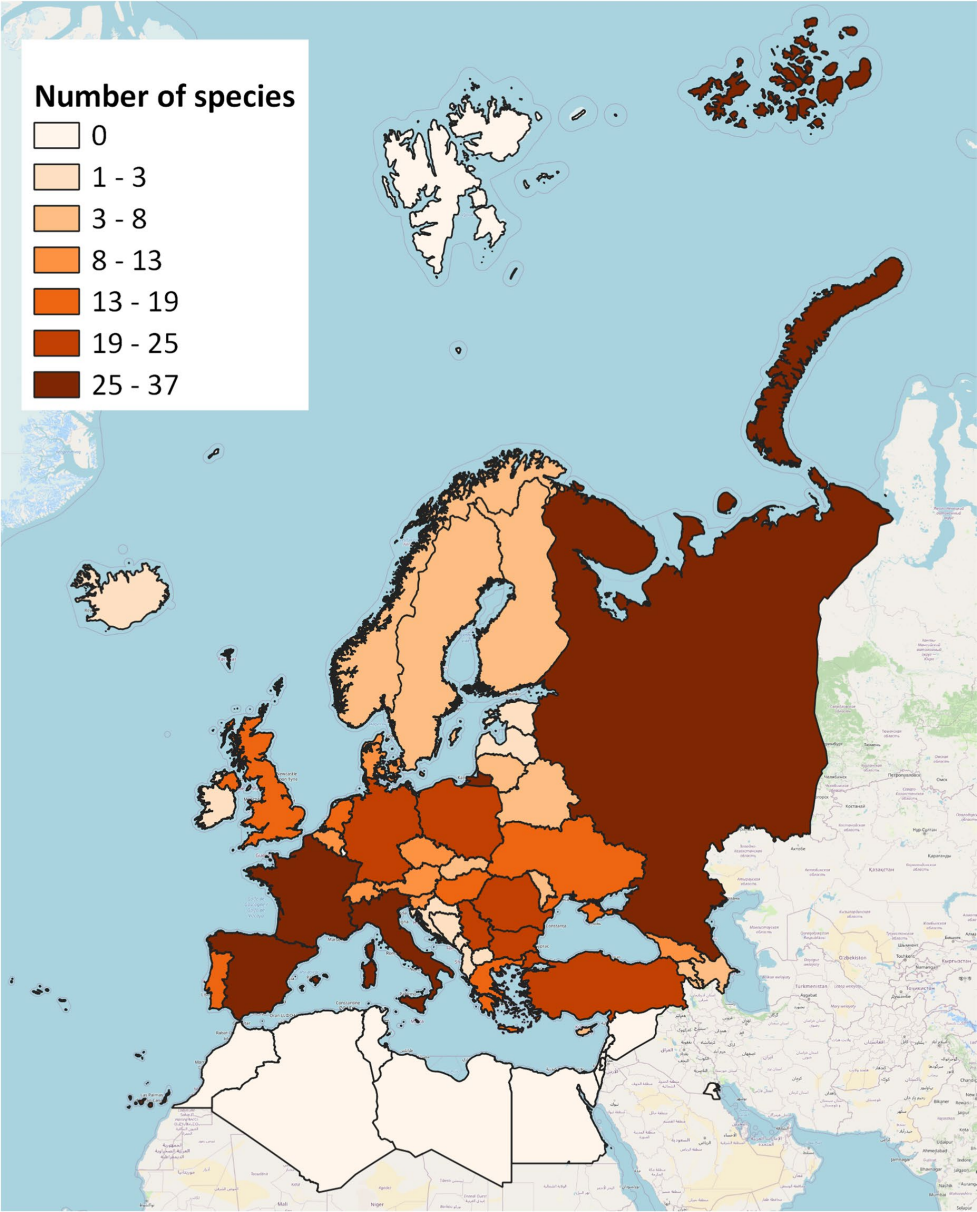
Example of coverage of geo-referenced records of bacteria with alien status across Europe (EPSG: 3857-WGS84/Pseudo Mercator, Contains Basemap: © OpenStreetMap).

The static version of the bacteria and viruses’ datasets (Tables 3, 4), as presented in this data descriptor, can be downloaded as.CSV format from *figshare*^34^. Two types of data are available for download: a dataset containing the information on the species taxonomy, origin, date of first introduction and pathways, and a dataset with species’ records at country scale. These primary datasets of alien bacteria and viruses were also integrated into the EASIN databases and services^35^, which ensure long-term persistence and preservation of data to support research communities on AS and research for policy. EASIN collects, manages and stores alien species data across Europe and makes them available through web tools for data analysis, open sharing and reporting. The quality of data inputs in EASIN is assured by thematic specialists^36^ (i.e. Editorial Board members and experts). Before being stored in the EASIN databases, the data is cleansed and standardised (spatial data accordingly to the Darwin Core Standard^37,38^). Data is released under the CC-BY licence enabling reuse with attribution to the original sources. The information on the bacteria and viruses’ invasion-related factors and location characteristics is stored in the EASIN Catalogue database (version 9.1), which currently hosts approximately 14,000 alien taxa in Europe. As an evolving data product, updates and corrections to bacteria and viruses’ data will occur with the following versions of the EASIN Catalogue. Versions are labelled using semantic versioning to indicate the introduced changes. The spatial data comprises polygon features at country scale (International Organization for Standardization (ISO) 3166-2) and 10 km × 10 km grid cells (EEA reference system) (Table 4, Fig. 5, and Supplementary Tables 3 and 4).

**Table 4:** Spatial occurrences of the bacteria and viruses datasets. Fields and description of the collected information for each species of bacteria and viruses.

Field	Description
SPECIES SCIENTIFIC NAME	Species scientific name
COUNTRY	ISO 3166-2 standard for occurrences at country scale
GRID	EEA reference code for 10 km × 10 km grid
YEAR	Year of the observation of the species
REFERENCE	reference to the publication for the species observation

### Data coverage

The number of bacteria and viruses (as of December 2021) is 446 including alien, cryptogenic and data deficient taxa. The main pathway of introduction is contaminant and escape (Fig. 4) for both bacteria (*e.g*. around 50% In Italy, Germany, France) and viruses (i.e. all taxa). Bacteria were also found to be introduced as stowaway (*e.g*. 25% in Netherlands), corridor (2–6%), release (2–6%) and unaided (2–5%). The rate of first records of bacteria in Europe decreased on average from 7.6 new records/year over 40 year period (1950–1990) to 5.6 records/year in 1991–2020 (Fig. 2a). For viruses, the rate of first records of introduction increased on average from 2.8 records/year (1950–1990) to 10.6 records/year in 1991–2020 (Fig. 2b). Finally, bacteria alien to Europe mainly originated from North and South America (around 80% taxa) and Americas and Asia for viruses. Information is still lacking for about 38% of viruses taxa.

## Technical Validation

The high-quality data on invasion-related factors and location characteristics is ensured by three strategies. First, invasion data were collected and overseen by experts from peer-reviewed scientific literature and intergovernmental organisations (*e.g*. OIE^30^, EFSA). Second, species occurrences were benchmarked against country-level information to ensure that the year of first introduction and later spread corresponded to governmental notification systems for pests and diseases (*e.g*. EPPO) and scientific findings (i.e. geographical records added to the taxa invasion-related factors).

Finally, a channel for user feedback is provided, where reporting of issues on the quality of single species information and occurrences is encouraged by using the EASIN Editorial Board^36^. This is a forum-like web interface allowing taxonomic experts to analyse registered users’ enquiries on species data and highlight records for revisions.

## Usage Notes

The datasets are available at *figshare*^34^ for download as compressed folder containing the static.CSV files for each group, *i.e*. bacteria and viruses. Species data can be accessed through the EASIN Catalogue and the ‘EASIN Species Search’ function which allows filtering single or groups of species by taxonomy, pathway, alien status and impact. Species occurrences can be mapped and downloaded from the EASIN Geodatabase using the ‘EASIN Species Mapper’ function which enables mapping species occurrences as polygons at country scale or at 10 km × 10 km cells. This tool allows also selecting occurrences for time ranges, and exporting the map in.PDF format or download it in WKT (EPSG:3035, ETRS89) or GeoJSON (EPSG:4326, WGS84) formats.

## .Supplementary information

Supplementary File 1

## Data Availability

No code is used in this study. Figures were produced using R statistical software^39^ and packages^40–42^.
