# Supplementary material for: European primary datasets of alien bacteria and viruses
Source: Sci Data. 2022 Jul 13;9:403. doi: 10.1038/s41597-022-01485-1 (PMC9279316; doi:10.1038/s41597-022-01485-1)
Supplement: Supplementary file 1 [file 41597_2022_1485_MOESM1_ESM.pdf]

Supplementary information

European primary datasets of alien bacteria and viruses

Magliozzi C et al.

Contents

Tables 2
Examples of pathways deduced from documented transmission routes of wild animal viruses 6

Tables

Table 1. *Categories and subcategories identified in the primary pathways of introduction of alien bacteria and viruses.*

**Category**	**Subcategory**
RELEASE IN NATURE	Biological control<br>Fishery in the wild<br>Hunting<br>Other intentional release
ESCAPE FROM CONFINEMENT	Agriculture<br>Aquaculture<br>Botanical garden or zoo or aquaria<br>Farmed animals<br>Forestry<br>Fur farms<br>Horticulture<br>Live food and live bait<br>Ornamental purpose other than horticulture<br>Other escape from confinement<br>Pet or aquarium or terrarium species<br>Research and ex-situ breeding<br>Biological control<br>Fishery in the wild<br>Hunting<br>Other intentional relase
TRANSPORT –CONTAMINANT	Contaminant nursery material<br>Contaminant on animals<br>Contaminant on plants<br>Food contaminant<br>Parasites on animals<br>Parasites on plants<br>Seed contaminant<br>Timber trade<br>Transportation of habitat material
TRANSPORT - STOWAWAY	Angling or fishing equipment<br>Container<br>Hitchhikers in or on airplane<br>Hitchhikers on ship or boat<br>Machinery or equipment<br>Organic packing material<br>Other means of transport<br>People and their luggage or equipment<br>Ship or boat ballast water<br>Ship or boat hull fouling<br>Vehicles
CORRIDOR	Interconnected waterways or basins or seas
UNAIDED	Natural dispersal across borders of IAS that have been introduced through previous pathways

Table 2. *Fields and descriptions of the dataset entries (Table 3 main text).*

Field	Description
COUNTRY OF FIRST INTRODUCTION IN EUROPE	AD:Andorra
COUNTRY OF FIRST INTRODUCTION IN EUROPE	AL:Albania
COUNTRY OF FIRST INTRODUCTION IN EUROPE	AM:Armenia
COUNTRY OF FIRST INTRODUCTION IN EUROPE	AT:Austria
COUNTRY OF FIRST INTRODUCTION IN EUROPE	AZ:Azerbaijan
COUNTRY OF FIRST INTRODUCTION IN EUROPE	BA:Bosnia and Herzegovina
COUNTRY OF FIRST INTRODUCTION IN EUROPE	BE:Belgium
COUNTRY OF FIRST INTRODUCTION IN EUROPE	BG:Bulgaria
COUNTRY OF FIRST INTRODUCTION IN EUROPE	BY:Belarus
COUNTRY OF FIRST INTRODUCTION IN EUROPE	CH:Switzerland
COUNTRY OF FIRST INTRODUCTION IN EUROPE	CY:Cyprus
COUNTRY OF FIRST INTRODUCTION IN EUROPE	CZ:Czechia
COUNTRY OF FIRST INTRODUCTION IN EUROPE	DE:Germany
COUNTRY OF FIRST INTRODUCTION IN EUROPE	DK:Denmark
COUNTRY OF FIRST INTRODUCTION IN EUROPE	DZ:Algeria
COUNTRY OF FIRST INTRODUCTION IN EUROPE	EE:Estonia
COUNTRY OF FIRST INTRODUCTION IN EUROPE	EG:Egypt
COUNTRY OF FIRST INTRODUCTION IN EUROPE	EL:Greece
COUNTRY OF FIRST INTRODUCTION IN EUROPE	ES:Spain
COUNTRY OF FIRST INTRODUCTION IN EUROPE	FI:Finland
COUNTRY OF FIRST INTRODUCTION IN EUROPE	FO:Faroes
COUNTRY OF FIRST INTRODUCTION IN EUROPE	FR:France
COUNTRY OF FIRST INTRODUCTION IN EUROPE	GE:Georgia
COUNTRY OF FIRST INTRODUCTION IN EUROPE	GG:Guernsey
COUNTRY OF FIRST INTRODUCTION IN EUROPE	GI:Gibraltar
COUNTRY OF FIRST INTRODUCTION IN EUROPE	HR:Croatia
COUNTRY OF FIRST INTRODUCTION IN EUROPE	HU:Hungary
COUNTRY OF FIRST INTRODUCTION IN EUROPE	IE:Ireland
COUNTRY OF FIRST INTRODUCTION IN EUROPE	IL:Israel
COUNTRY OF FIRST INTRODUCTION IN EUROPE	IM:Isle of Man
COUNTRY OF FIRST INTRODUCTION IN EUROPE	IS:Iceland
COUNTRY OF FIRST INTRODUCTION IN EUROPE	IT:Italy
COUNTRY OF FIRST INTRODUCTION IN EUROPE	JE:Jersey
COUNTRY OF FIRST INTRODUCTION IN EUROPE	LB:Lebanon
COUNTRY OF FIRST INTRODUCTION IN EUROPE	LI:Liechtenstein
COUNTRY OF FIRST INTRODUCTION IN EUROPE	LT:Lithuania
COUNTRY OF FIRST INTRODUCTION IN EUROPE	LU:Luxembourg
COUNTRY OF FIRST INTRODUCTION IN EUROPE	LV:Latvia
COUNTRY OF FIRST INTRODUCTION IN EUROPE	LY:Libya
COUNTRY OF FIRST INTRODUCTION IN EUROPE	MA:Morocco
COUNTRY OF FIRST INTRODUCTION IN EUROPE	MC:Monaco
COUNTRY OF FIRST INTRODUCTION IN EUROPE	MD:Moldova
COUNTRY OF FIRST INTRODUCTION IN EUROPE	ME:Montenegro

COUNTRY OF FIRST INTRODUCTION IN EUROPE	MK:North Macedonia
COUNTRY OF FIRST INTRODUCTION IN EUROPE	MT:Malta
COUNTRY OF FIRST INTRODUCTION IN EUROPE	NL:Netherlands
COUNTRY OF FIRST INTRODUCTION IN EUROPE	NO:Norway
COUNTRY OF FIRST INTRODUCTION IN EUROPE	PL:Poland
COUNTRY OF FIRST INTRODUCTION IN EUROPE	PS:Palestine
COUNTRY OF FIRST INTRODUCTION IN EUROPE	PT:Portugal
COUNTRY OF FIRST INTRODUCTION IN EUROPE	RO:Romania
COUNTRY OF FIRST INTRODUCTION IN EUROPE	RS:Serbia
COUNTRY OF FIRST INTRODUCTION IN EUROPE	RU:Russian Federation
COUNTRY OF FIRST INTRODUCTION IN EUROPE	SE:Sweden
COUNTRY OF FIRST INTRODUCTION IN EUROPE	SI:Slovenia
COUNTRY OF FIRST INTRODUCTION IN EUROPE	SJ:Svalbard and Jan Mayen
COUNTRY OF FIRST INTRODUCTION IN EUROPE	SK:Slovakia
COUNTRY OF FIRST INTRODUCTION IN EUROPE	SM:San Marino
COUNTRY OF FIRST INTRODUCTION IN EUROPE	SY:Syria
COUNTRY OF FIRST INTRODUCTION IN EUROPE	TN:Tunisia
COUNTRY OF FIRST INTRODUCTION IN EUROPE	TR:Turkey
COUNTRY OF FIRST INTRODUCTION IN EUROPE	UA:Ukraine
COUNTRY OF FIRST INTRODUCTION IN EUROPE	UK:United Kingdom
COUNTRY OF FIRST INTRODUCTION IN EUROPE	VA:Vatican City
**ORIGIN (TERRESTRIAL/FRESHWATER)**	
America	
America North	
America South	
America Central	
Asia	
Asia North	
Asia South	
Asia East	
Asia West	
Asia Central	
Asia North-East	
Asia North-West	
Asia South-East	
Asia South-West	
Indian Subcontinent	
Ponto-Caspian	
Europe	
Europe North	
Europe South	
Africa	
Africa North	

Africa South	
Africa East	
Africa West	
Africa Central	
Oceania	
Australia	
New Zealand	
Arctic zone	
Antartic zone	
Polar regions	
Temperate regions	
Circumtropical	
Cosmopolitan	
Unknown	
**ORIGIN (MARINE)**	
Arctic	
Baltic Sea	
Black Sea	
Central Indo-Pacific	
Eastern Indo-Pacific	
Mediterranean Sea	
North-East Atlantic Ocean	
Saharan upwelling	
Southern Ocean	
Temperate Australasia	
Temperate Northern Pacific	
Temperate Northwest Atlantic	
Temperate South America	
Temperate Southern Africa	
Tropical Atlantic	
Tropical Eastern Pacific	
Western Indo-Pacific	
Unknown	

Table 3. *Alien bacteria for which geospatial information is not yet available.*

Pantoea stewartii subsp. *stewartii*

Table 4. *Alien viruses for which geospatial information is not yet available.*

Groundnut ringspot orthotospovirus
Hibiscus latent ringspot virus
Honeysuckle yellow vein mosaic betasatellite
Honeysuckle yellow vein mosaic virus
Honeysuckle yellow vein virus
Opuntia virus X

Potato yellow vein virus
Pothos latent virus
Sweet potato leaf curl deltasatellite 2
Sweet potato leaf curl deltasatellite 3

Examples of pathways deduced from documented transmission routes of wild animal viruses

African swine fever virus: Transmission by soft ticks in African *suids*, and (as far as we know) only by direct of indirect contact (contaminated commodities, meat…) in Europe. Transmitted probably by contaminated feed (garbage, game feeding) to free living wild-boars and spread among them by contagion. Pathway: the current *epizootic* is believed to have been introduced by contaminated commodities (illegal trade) in Georgia during the early years 2000 then spread to wild-boars at the border with Russia, and spread throughout Russia up to European Eastern countries by both routes, i.e. large long distance movements by illegal trade, or accidental spread of contaminated food or feed in domestic pigs then local spread by contagion in free living populations of wild-boars. Pathway: illegal international movements of contaminated food, feed or commodities, trans-border transmission by contagion in free living wild boars.

Alphainfluenzavirus (Avian influenza) transmitted by direct (beak to beak) or indirect (contaminated soil or water) contact. Pathway: natural or human induced movements of infected birds shedding virus.

Avian orthoavulavirus 1 (Newcastle disease) As influenza

Avian pox of Tits: Highly contagious virus common to various species of birds, probably spread to tits by healthy spreaders and locally amplified by artificial feeding. Pathway: still unknown, probably bird movements.

Beak and feather disease virus: Transmitted by direct or indirect contact. Pathway: trade of zoo or pet psittacids (global spread) followed by local maintenance (in Europe) of feral psittacids.

Bluetongue virus: Transmitted by bites of blood sucking midges (*Culicoides* sp.) Pathway: spread of infected midges, and local diffusion.

Cyprinid herpesvirus 3: highly contagious virus spread by infected healthy cyprinid, carps included, transmitted from infected to susceptible by direct contact or waterborne exposure. Pathway: trade and movements of fishes released in surface waters and ponds.

European brown hare syndrome virus: As RHD (below), supposed to have evolved after contamination of hares by rabbit viruses. Pathway: trans-border trade or movement of contaminated hares (game release) has been documented, then local spread by contagion.

Pestivirus D: Transmitted by direct contacts between infected and susceptible chamois. Pathway: Still unknown, supposed to have resulted from adaptation of sheep virus transmitted to chamois.

Rabbit hemorrhagic disease virus: Transmitted by direct contact (highly contagious) Pathway not yet clarified. Probable passage from domestic infected rabbits to free living rabbits and vice et versa. Pathway: Supposed to have been (re) introduced from China to Europe by trade.

Ranavirus: Transmitted by multiple routes (direct or indirect contact), including contaminated soil, waterborne exposure, and ingestion of infected tissues. Virus originated from fish infections and has been adapted to amphibians and reptiles. Pathway Global and local release of infected fishes in waters for fishing purpose, contaminated waters, release of pets in the wild, contaminated materials or equipment immersed in waters.

Squirrel pox virus: direct or indirect contact between susceptible European red squirrels and invasive infected gray squirrels. The last one being healthy spreader and maintenance host of the infection. Pathway: introduction in Europe of infected American gray squirrels during the XIXth century, spread of invasive Gray populations, progressive eradication of the Red by competition and disease mediated mortality.

Usutu virus: as West-Nile (same group of viruses, similar transmission).

West nile virus: Transmitted by mosquitoes and ticks (ornithophilous or ubiquitous).

Pathway: natural movements of infected birds and or, their vectors.
